# A Unique Case of Goodpasture’s Syndrome-Induced Cardiorenal Syndrome

**DOI:** 10.7759/cureus.64269

**Published:** 2024-07-10

**Authors:** Chidambaram Chinniah, Alexander Pyronneau, Gauthier Stepman, Rias Ali

**Affiliations:** 1 Cardiology, HCA Healthcare/University of South Florida Morsani College of Medicine, Graduate Medical Education at HCA Florida Trinity Hospital, Trinity, USA; 2 Internal Medicine, HCA Healthcare/University of South Florida Morsani College of Medicine, Graduate Medical Education at HCA Florida Trinity Hospital, Trinity, USA

**Keywords:** dilated cardiomyopathy, congestive heart failure, anti-gbm disease, automated implantable cardiac defibrillator (aicd), goodpasture's syndrome, cardiorenal syndrome

## Abstract

Goodpasture's syndrome (GPS) is a rare small vessel vasculitis characterized by circulating antibodies directed against the glomerular and alveolar basement membrane leading to renal and pulmonary manifestations. Here, we discuss a unique case of a 30-year-old Caucasian male smoker initially presenting with hemoptysis and anemia who was found to have biopsy-proven GPS with elevated anti-glomerular basement membrane (anti-GBM) antibodies. Unfortunately, the patient failed four months of standard treatment for GPS leading to end-stage renal disease (ESRD), while uniquely developing cardiorenal syndrome (CRS) with non-ischemic cardiomyopathy resulting in systolic and diastolic heart failure (HF). Despite aggressive medical management and hemodialysis, the patient’s cardiac function continued to decline and the decision was made to insert an automatic implantable cardioverter defibrillator (AICD). To our knowledge, this is the first reported case of an anti-GBM-positive GPS patient who developed dilated cardiomyopathy. The importance of this report is to illustrate the rarity of developing CRS with non-ischemic cardiomyopathy and congestive heart failure from GPS and highlight the difficulty of determining management changes beyond guideline-directed medical therapy (GDMT) in GPS to slow the progression of worsening cardiac function.

## Introduction

Goodpasture's syndrome (GPS), also known as anti-glomerular basement membrane (anti-GBM) disease, is an autoimmune vasculitis of small vessels characterized by circulating antibodies directed against the glomerular and alveolar basement membrane [1]. GPS is a rare disorder with less than two cases per million patients, most commonly seen in the White population with a peak incidence in the 3rd and 6th-7th decades of life [2]. Although most cases of GPS have an unclear etiology, proposed environmental triggers include kidney injury (e.g., lithotripsy, ureteral obstruction, and membranous nephropathy), pulmonary injury (e.g., pulmonary infections and smoking), and immune dysregulation (e.g., HIV) [3]. GPS often results in rapidly progressive glomerulonephritis and/or alveolar hemorrhage accounting for 10%-15% of all cases of crescentic glomerulonephritis [4], with only very few cases progressing to end-stage renal disease (ESRD) [5]. Patients less than 30 years of age are more likely to be male and present with pulmonary hemorrhage whereas older patients (>50 years) are more likely to be female and present with isolated glomerulonephritis [6,7]. The treatment strategy of GPS includes plasmapheresis, to remove circulating anti-GBM antibodies, as well as anti-inflammatory medications (i.e., glucocorticoids) and immunosuppression therapy (i.e., cyclophosphamide), to minimize new antibody formation [8].

Here, we present a case of a 30-year-old Caucasian male smoker who was diagnosed with anti-GBM-positive GPS, failed four months of therapy (e.g., plasmapheresis, steroids, and cyclophosphamide), and developed ESRD. The patient subsequently developed cardiorenal syndrome (CRS) with non-ischemic cardiomyopathy and recurrent pleural effusions secondary to congestive heart failure (CHF), which required an automated implantable cardiac defibrillator (AICD) placement and a PleurX catheter (Becton, Dickinson and Company, Franklin Lakes, NJ). To our knowledge, this is the first reported case of an anti-GBM-positive GPS patient who developed dilated cardiomyopathy. Our finding that CRS with non-ischemic cardiomyopathy and CHF developed in a patient diagnosed with GPS highlights the possibility that GPS, when refractory to treatment, may play a critical role in worsening cardiac function in select cases.

## Case presentation

**Figure 1:**
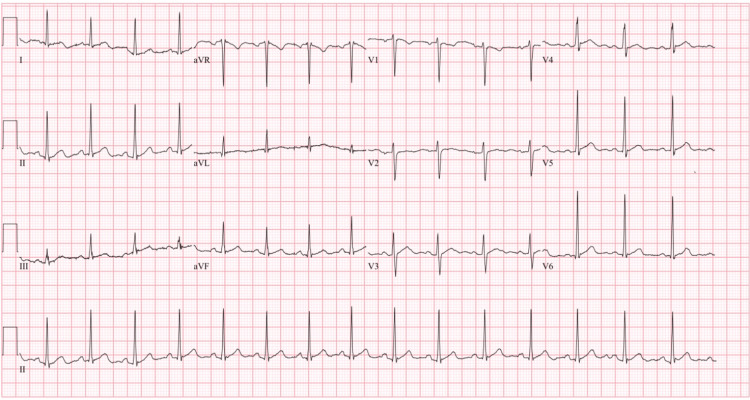
EKG on initial presentation. EKG on initial presentation shows normal sinus rhythm with no evidence of acute ischemia.

The patient was a 30-year-old male smoker who first presented to the emergency department with a six-month history of hemoptysis, which progressively worsened one week prior to presentation, in the setting of shortness of breath. Upon initial lab work, he was found to have anemia and chronic kidney disease stage 5 (CKD5: creatinine of 5.4 mg/dL and glomerular filtration rate of 12.6 mL/min/1.73 m^2^ with only prior labs showing normal baseline renal function with a creatinine of 1.2 mg/dL 12 years prior to the initial presentation). At the initial presentation, troponins were within normal limits and an EKG showed normal sinus rhythm with no evidence of acute ischemia (Figure 1). Nephrology and pulmonology were consulted. Workup demonstrated marked elevation of serum anti-GBM antibodies and a kidney biopsy demonstrated extensive crescentic and necrotizing glomerulonephritis, along with strong linear glomerular capillary loop staining for IgG by immunofluorescence consistent with anti-GBM glomerulonephritis. Of note, antinuclear antibody (ANA), cytoplasmic antineutrophil cytoplasmic autoantibody (c-ANCA), and perinuclear-antineutrophil cytoplasmic autoantibody (p-ANCA) were negative. This confirmed the patient’s suspected GPS and plasmapheresis, steroids, and cyclophosphamide were started. The patient also started hemodialysis (HD) during the initial hospitalization and was scheduled to continue outpatient HD sessions.

**Figure 2:**
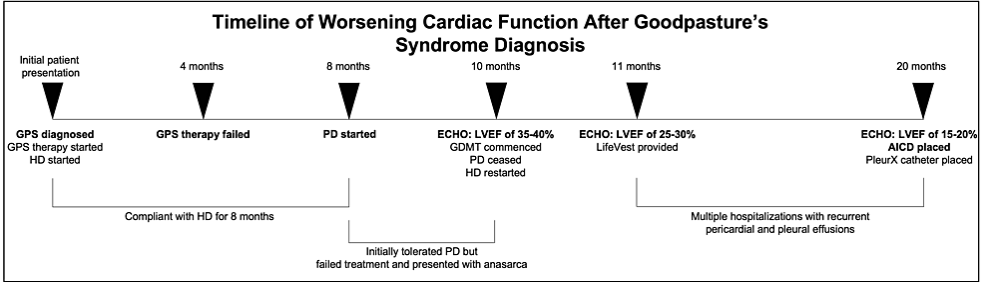
Timeline of worsening cardiac function after Goodpasture’s syndrome diagnosis. On initial presentation, the patient was diagnosed with biopsy-proven Goodpasture’s syndrome (GPS) and started on standard GPS therapy (e.g., plasmapheresis, steroids, and cyclophosphamide) and hemodialysis (HD). The patient failed after four months of treatment since renal function did not recover and thus the patient continued HD sessions. The patient was compliant with HD sessions three times per week until eight months after the initial presentation, whereby the patient elected to try peritoneal dialysis (PD) but eventually failed PD treatment. This led to anasarca and a new diagnosis of systolic heart failure 10 months after the initial presentation. Although PD ceased and HD was reinitiated at that time, the patient’s cardiac function continued to worsen over the next several months until the patient’s ejection fraction was 15-20% 20 months after the initial presentation, which led the patient to get an automatic implantable cardioverter defibrillator (AICD). GPS: Goodpasture’s syndrome; HD: hemodialysis; PD: peritoneal dialysis; ECHO: echocardiogram; GDMT: guideline-directed medical therapy; LVEF: left ventricular ejection fraction; AICD: automatic implantable cardioverter defibrillator.

Upon discharge, the patient received plasmapheresis, steroids, and cyclophosphamide for four months, yet the patient failed treatment as their renal function did not recover. The patient was compliant with three HD sessions per week for approximately eight months and elected to switch to peritoneal dialysis (PD) for greater schedule flexibility. The patient initially tolerated PD for a few weeks until PD treatment failed 10 months after his initial hospitalization. He subsequently presented with worsening shortness of breath and edema (Figure 2).

**Figure 3:**
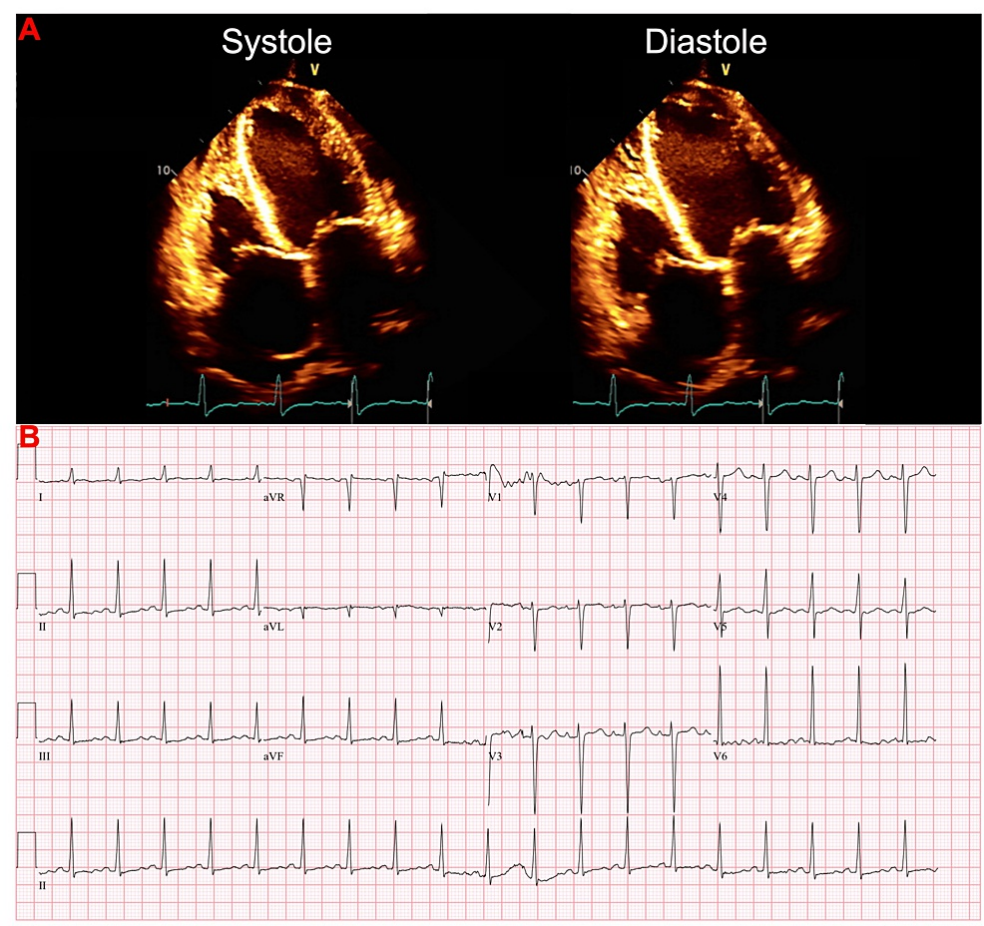
Initial echocardiogram and corresponding EKG 10 months after Goodpasture’s syndrome diagnosis. (A) 2D echocardiography showing left ventricular mild to moderate dilation with a markedly reduced ejection fraction of 35% to 40%. There was also markedly diffuse hypokinesis, and grade 2 diastolic dysfunction (G2DD). The left atrium and right atrium were moderately dilated and the right ventricle was mildly to moderately dilated with mildly reduced systolic function. There was moderate to severe mitral valve regurgitation, moderate tricuspid valve regurgitation, and mild aortic valve regurgitation. The inferior vena cava was mildly dilated, and the systolic pressure of the pulmonary arteries was moderately increased. (B) EKG 10 months after initial presentation showed sinus tachycardia, nonspecific T-wave abnormalities, and prolonged QTc (491 ms).

Physical exam was significant for anasarca (bilateral lower extremity edema up to the knees, scrotal swelling, and ascites). N-terminal pro-B-type natriuretic peptide (NT-proBNP) was greater than 175,000 pg/mL and high-sensitivity troponins were elevated at 428 ng/L, most likely secondary to demand ischemia and impaired renal clearance. Initial echocardiogram showed a left ventricular ejection fraction (LVEF) of 35-40% with moderate to marked diffuse hypokinesis and grade 2 diastolic dysfunction (G2DD), moderate to severe mitral valve regurgitation, and a small pericardial effusion (Figure 3A). EKG showed sinus tachycardia with nonspecific T-wave abnormalities (Figure 3B). The decision was made to cease PD, reinitiate HD, and begin guideline-directed medical therapy (GDMT), which initially included metoprolol succinate and lisinopril. After trialing many heart failure medications, he was eventually medically optimized and treated with metoprolol succinate, valsartan, and spironolactone.

**Figure 4:**
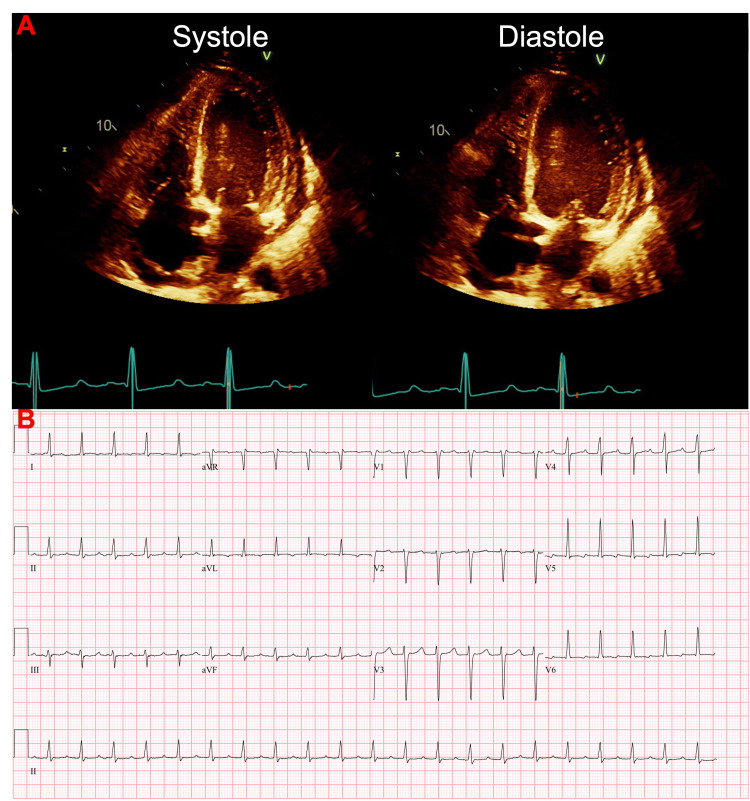
Worsening cardiac function in the setting of accelerated junctional rhythm 11 months after initial presentation. (A) 2D echocardiography showing left ventricular moderate dilation with severely reduced ejection fraction of 25% to 30%. There was severely diffuse hypokinesis, a moderate increase in wall thickness, moderate concentric hypertrophy, and grade 3 diastolic dysfunction (G3DD). The left atrium was moderately dilated. The right atrium was markedly dilated yet the right ventricle was normal in size and normal in systolic function. There was mild to moderate mitral valve regurgitation, moderate to severe tricuspid valve regurgitation, and trivial aortic valve regurgitation. The inferior vena cava was mildly dilated, and the systolic pressure of the pulmonary arteries was moderately increased. (B) EKG 11 months after initial presentation showed accelerated junctional rhythm with an elevated QTc (501 ms).

Within one month (11 months after the initial presentation), the patient presented back to the hospital with palpitations. A repeat echocardiogram showed an LVEF of 25-30%, grade 3 diastolic dysfunction (G3DD), severe diffuse hypokinesis, and moderate concentric hypertrophy (Figure 4A). EKG showed an accelerated junctional rhythm (Figure 4B) and high-sensitivity troponins were elevated (310 ng/L and 291 ng/L). After the patient was stabilized, the decision was made to give the patient a LifeVest (ZOLL Medical Corporation, Chelmsford, MA).

**Figure 5:**
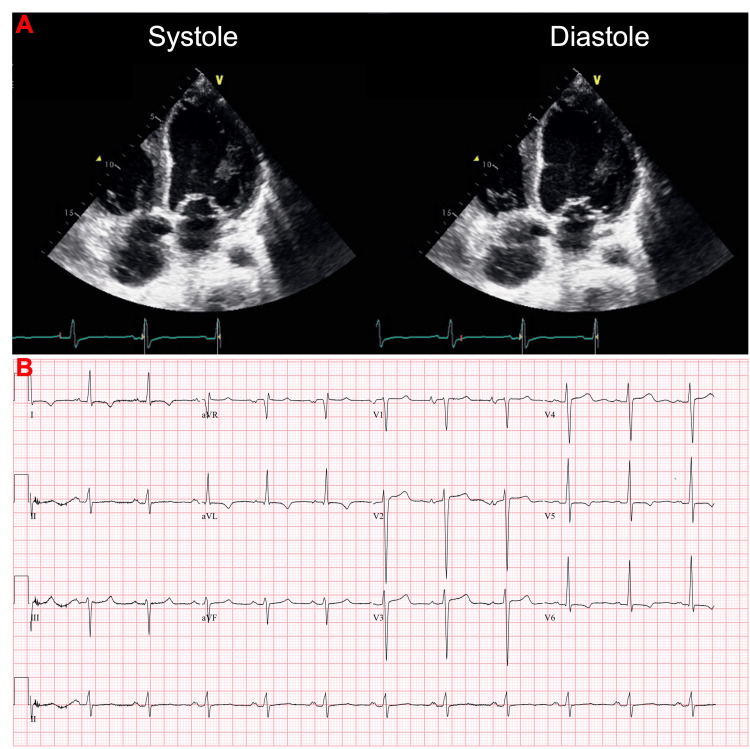
Severely reduced systolic function 20 months after initial presentation. (A) 2D echocardiography shows left ventricular moderate dilation with severely reduced ejection fraction of 15% to 20%. There was severely diffuse hypokinesis, grade 2 diastolic dysfunction (G2DD), and no regional wall motion abnormalities. The left atrium and right atrium were normal in size, and the right ventricular size and systolic function were normal. There was moderate mitral valve regurgitation and no tricuspid or aortic valve regurgitation. The inferior vena cava was dilated, and the systolic pressure of the pulmonary arteries was within the normal range. (B) EKG 20 months after the initial presentation shows normal sinus rhythm with possible left atrial enlargement, left ventricular hypertrophy criteria, T-wave abnormalities suggestive of possible lateral ischemia, and prolonged QTc (467 ms).

The patient remained compliant with HD sessions over the next few months; however, the patient frequently returned to the hospital with shortness of breath and was found to have recurrent pericardial effusions and pleural effusions (Figure 2). A thoracentesis was performed multiple times during these hospitalizations and studies of the pleural fluid were transudative in nature. Twenty months after the initial hospitalization, the patient presented again with worsening shortness of breath. An echocardiogram at that time showed LVEF of 15-20% and dilated cardiomyopathy (Figure 5). EKG showed normal sinus rhythm with possible left atrial enlargement, left ventricular hypertrophy, T-wave abnormalities suggestive of possible lateral ischemia, and a prolonged QTc (467ms), with elevated high sensitivity troponins (1007 ng/L, 960 ng/L, 998 ng/L, and 1223 ng/L).

**Figure 6:**
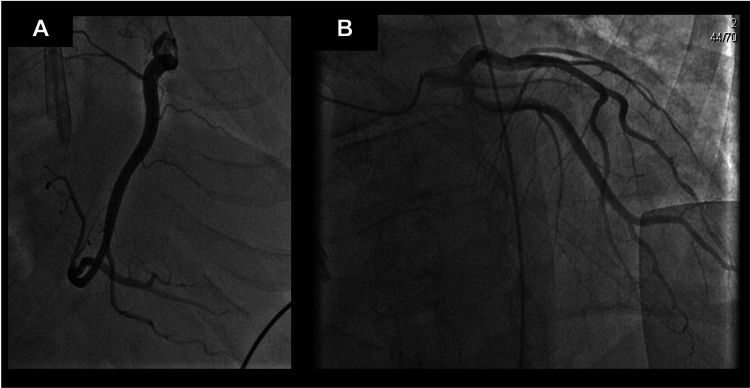
Left heart catheterization 20 months after initial presentation displays nonischemic dilated cardiomyopathy. (A) The right coronary artery (RCA) displayed is a moderate-sized dominant vessel giving off multiple branches. The contours of the RCA are smooth without intraluminal narrowing and normal flow. (B) The left main coronary artery is a small to moderate-sized vessel in which the vessel walls are smooth and the contours are smooth. The left anterior descending coronary artery and the left circumflex artery are small-to-moderate caliber vessels with no significant intraluminal narrowing with contours of the vessels being smooth with normal flow. The left ventricular ejection fraction was 15% with diastolic and systolic dysfunction.

A left heart catheterization confirmed non-ischemic cardiomyopathy, with an LVEF of 15% (Figure 6). Additionally, during this hospitalization, pericardial effusion and large pleural effusion were noted, which required a subxiphoid pericardial window and PleurX (Becton, Dickinson and Company, Franklin Lakes, NJ) insertion by cardiothoracic surgery. Given the patient’s worsening cardiac function, the decision was made to place an AICD.

## Discussion

Here, we demonstrate a unique case of GPS-related pathology resulting in CRS. CRS is defined as the collective dysfunction of the heart and the kidneys resulting in a cascade of feedback mechanisms causing damage to both organs [9]. CRS is subdivided into five subcategories based on the etiology, pathophysiology, duration, and pattern of cardiac and renal dysfunction [10]. Type 1 CRS (also called acute CRS) occurs when rapid worsening of cardiac function results in acute kidney injury, type 2 CRS (also called chronic CRS) occurs when chronic abnormalities in cardiac function lead to chronic kidney disease (CKD), type 3 CRS (also called acute renocardiac syndrome) occurs when rapid worsening of kidney function leads to acute cardiac dysfunction, type 4 CRS (also called chronic renocardiac syndrome) occurs when progressive CKD leads to worsening cardiac function, and type 5 CRS (also known as secondary CRS) occurs when a systemic illness causes combined cardiac and renal dysfunction [9]. Based on our findings, we suspect that our patient most likely displayed type 4 CRS whereby progressive CKD led to worsening cardiac function. An otherwise healthy 30-year-old male presented with six months of hemoptysis whereby workup was significant for CKD5 and biopsy-proven GPS. Despite four months of standard GPS therapy utilizing plasmapheresis, steroids, and cyclophosphamide, the patient’s renal function did not recover and thus the patient required continued HD due to developing ESRD. The patient was compliant with HD sessions and did not display signs of fluid overload until he elected to switch to PD eight months after the initial presentation. Even though the patient was reinitiated on HD 10 months after the initial presentation (Figure 2), his cardiac function continued to decline in subsequent months, while frequently being fluid-overloaded requiring multiple thoracenteses. We propose that ESRD from refractory GPS in combination with inadequate fluid removal was the major contributor that caused CRS and the patient’s heart failure.

The pathophysiology underlying CRS is complex and incompletely understood. The major pathophysiological factors of CRS include elevated central venous and abdominal pressures, decreased cardiac output and cardiac index, neurohormonal dysregulation (renin-angiotensin-aldosterone system (RAAS) activation, sympathetic nervous system activation, and vasopressin), oxidative stress, inflammatory mediators, renal failure-related disturbances, and iatrogenic factors [10]. More specifically, vasoconstriction, sodium and water reabsorption, oxidative stress, RAAS, and sympathetic nervous system (SNS) activation have been proposed to contribute to type 4 CRS [10], which we suspect our patient most likely had. A plausible scenario in our patient is that refractory GPS caused CKD, which in turn led to ESRD and thus promoted sodium/water reabsorption, RAAS/SNS activation, vasoconstriction, and oxidative stress that precipitated CRS, thus leading to nonischemic cardiomyopathy and CHF. It is unclear why more patients who progress to ESRD from refractory GPS do not develop CRS; however, variability in environmental triggers (i.e., smoking history in our patient), genetic factors (i.e., the growing association of human leukocyte antigens between dilated cardiomyopathy and GPS [11,12]), and/or clinical features exhibited may play an important role.

Although we suspect GPS pathology is a major contributor to the patient’s CRS and declining cardiac function, we cannot exclude other possible causes of the patient’s heart failure. The first potential contributing factor includes cyclophosphamide, a known cardiotoxic medication with common manifestations, including heart failure, myocarditis, tachyarrhythmias, hypotension, and pericardial disease [13]. We cannot completely rule out the possibility that cyclophosphamide contributed to the patient’s heart failure as the patient received cyclophosphamide for four months after the diagnosis of GPS was made. Of note, the cardiotoxic side effects of cyclophosphamide typically present within the first 48 hours of drug administration and may be seen up to 10 days after initiation [13]. Since the patient tolerated four months of treatment with cyclophosphamide, and the patient’s cardiac function was impaired 10 months after the initial presentation (Figure 2), it is possible yet less likely that the patient had severe cardiotoxicity to cyclophosphamide as he might have manifested heart failure symptoms earlier.

A second potential contributing factor to the patient’s heart failure is myocarditis. Myocarditis is an inflammatory disease of the myocardium [14]. The main cause of myocarditis is viral infection but other causes can include non-viral infections, toxins, medications, vaccines, and autoimmune disease [15]. Myocarditis can have a variety of clinical manifestations such as chest pain, new or worsening heart failure, chronic heart failure, life-threatening hemodynamic compromise (i.e., fulminant myocarditis, with cardiogenic shock and severely reduced LVEF), life-threatening arrhythmias, or conduction disturbances [15]. Diagnostic tools used when myocarditis is suspected include cardiac MRI, which has the highest sensitivity if performed two to three weeks after the initial clinical presentation, endomyocardial biopsy, which remains the gold-standard technique to diagnose acute myocarditis [16] and reveals an inflammatory infiltrate with necrosis or degeneration of adjacent myocytes, markers of myocyte injury and inflammation such as erythrocyte sedimentation rate (ESR) and C-reactive protein (CRP) although not specific and not necessarily increased in myocarditis, high sensitivity troponins, brain natriuretic peptide (BNP) and NT-proBNP (useful but not specific) [15]. Ten months after the initial presentation, the patient presented with new-onset heart failure in the setting of fluid overload with failed peritoneal dialysis two months prior (eight months after the initial presentation). We cannot completely rule out the possibility that myocarditis contributed to the patient’s new heart failure. Our facility lacks a cardiac MRI, which would have been the best test to rule out myocarditis at that time. Also, endomyocardial biopsy was not indicated at the time, especially since the patient did not show signs of inflammation. NT-proBNP and high-sensitivity troponins were elevated 10, 11, and 20 months after the initial presentation. However, we suspect the initial elevation of NT-proBNP was secondary to GPS-induced CRS in combination with improper fluid removal, and the elevated high-sensitivity troponins were secondary to demand ischemia and impaired renal clearance as the patient rarely complained of chest pain during these hospitalizations. It is important to note that fulminant myocarditis, an acute form of myocarditis, is characterized by a severe and rapid decline in cardiac function leading to hemodynamic instability within two weeks that can occur within two or three days [17]. Patients often display a sharp drop in blood pressure that is maintained by vasoactive drugs and/or require mechanical circulation support devices as cases can be fatal [17]. Since our patient’s decline in cardiac function was relatively gradual (10-11 months after the initial hospitalization), fulminant myocarditis may be a potential contributor, but less likely. Furthermore, the patient did not require vasoactive drugs and/or mechanical circulation support and the patient recovered well after each hospitalization. Nonetheless, although rare, since the cardiac MRI or endomyocardial biopsy was not performed, we cannot completely rule out myocarditis as a potential contributor to the patient’s heart failure.

A third potential contributing factor to the patient’s heart failure is vasculitis. Systemic vasculitides are complex clinical syndromes characterized by fibrinoid necrosis and inflammation associated with endothelial cell activation of blood vessels and vascular remodeling that can affect any organ with heterogeneous clinical manifestations [18]. The vasculitides can involve arteries of all sizes and some cases can involve aneurysm development and dilation or narrowing and vessel occlusion [18]. Thus, all primary vasculitides can potentially target the heart and cause cardiac dysfunction [19]. Some of these vasculitides include granulomatosis with polyangiitis, microscopic polyangiitis, eosinophilic granulomatosis with polyangiitis, and polyarteritis nodosa [18,19]. Many of these vasculitides, which cause heart failure, are antineutrophil cytoplasmic antibody (ANCA)-associated [20]. On initial presentation, our patient was negative for ANA, c-ANCA, and p-ANCA. At first glance this may rule out vasculitides; however, there are also cases of ANCA-negative vasculitides that display cardiac dysfunction and heart failure [21]. In these cases, a definitive diagnosis would include endomyocardial biopsy in combination with cardiac MRI [21]. Although extremely rare, since a cardiac MRI and/or endomyocardial biopsy was not performed, we cannot completely rule out vasculitis involvement to explain the patient’s heart failure.

GPS is rare and there is scarce literature on subsequent cardiac manifestations [12,22,23], especially after failed GPS treatment. Moreover, to our knowledge, this is the first case to demonstrate anti-GBM-positive GPS concurrently with dilated cardiomyopathy. Given how few cases there are, it is hard to determine whether GPS-related cardiomyopathy is generally unresponsive to GDMT or if this was unique to the patient’s presentation. Therefore, emphasis should target early diagnosis and intervention, which are essential to therapy response and the long-term prognosis of GPS and subsequent GPS-related cardiomyopathy. Had therapy started sooner, a different outcome may have occurred for this patient. However, the outcome of our patient’s case was less favorable given how advanced the disease was on presentation. GPS therapy includes plasmapheresis to remove circulating anti-GBM antibodies, as well as steroids and cytotoxic drugs to minimize new antibody formation [1], where less than one-third of patients retain renal function after six months of follow-up [24]. Our patient failed after four months of initial therapy. An important takeaway from this case is the possibility of using alternative therapeutic strategies for refractory GPS before ESRD develops to protect cardiac function. For instance, an important medication, which has shown great promise in refractory GPS is rituximab [25]. Rituximab is a monoclonal antibody against CD20 on B cells, which has shown success in multiple autoimmune and neoplastic conditions [25]. In GPS, multiple studies, where rituximab was used either as a first-line therapy or an addition to standard therapy, have shown variable success [26-28] yet the evidence is scarce. Another therapy, which has shown great promise and is currently being investigated in rare IgG autoimmune conditions and transplant rejection, is imlifidase. Imlifidase is a cysteine protease that cleaves IgG [29] and has been shown in an anti-GBM mouse model to reduce proteinuria and degrade both circulating and kidney-bound anti-GBM antibodies [30]. One study utilized a single dose of imlifidase in combination with corticosteroids, cyclophosphamide, and plasmapheresis in 15 GPS patients and found that six hours after imlifidase administration, antibodies were either undetectable or within the reference range [29]. Moreover, six months after administration, 10 patients were dialysis independent, one died, and four had kidney failure requiring dialysis [29]. Thus, future studies should explore the use of alternative strategies in addition to GPS standard therapy, to preserve renal function and delay the progression to renal failure and possible CRS.

## Conclusions

GPS is a rare disease that can progress to ESRD causing subsequent cardiorenal syndrome resulting in non-ischemic cardiomyopathy requiring a LifeVest or AICD placement. The prognosis remains poor; however, given how rare these cases are, it is difficult to determine management changes beyond GDMT in slowing or preventing worsening heart function and the need for AICD.
